# The Key Role of Personality Functioning in Understanding the Link Between Adverse Childhood Experiences and Loneliness: A Cross-Sectional Mediation Analysis

**DOI:** 10.3390/brainsci15060551

**Published:** 2025-05-23

**Authors:** Jeff Maerz, Roberto Viviani, Karin Labek

**Affiliations:** 1 Institute of Psychology, University of Innsbruck, 6020 Innsbruck, Austria; 2 Department of Psychiatry and Psychotherapy III, University of Ulm, 89075 Ulm, Germany

**Keywords:** personality functioning, adverse childhood experiences, ACE, loneliness, attachment, mediation

## Abstract

Background and Aims: Loneliness represents a critical public health concern, significantly affecting mental and physical health. Adverse childhood experiences (ACEs) have been recognized as predictors of loneliness, yet the underlying mechanisms remain unclear. The level of personality functioning, determined by self- and interpersonal impairments, has been proposed as a potential mediator but has not been empirically explored in this context. This study examined whether personality functioning mediates the relationship between cumulative and specific types of ACEs and loneliness using a cross-sectional design. Methods: An online survey of 334 participants (mean age = 25.96; 65% female) were assessed for ACEs using a modified version of the KERF-40-I scale, their personality functioning using the Level of Personality Functioning Scale (LPFS-BF), and loneliness using the revised UCLA Loneliness Scale. Results: Higher ACE exposure was significantly associated with loneliness and greater impairments in overall personality functioning and self-functioning. After controlling for other ACE subtypes, emotional neglect remained associated with the overall level of personality functioning, while emotional neglect and sexual violence were associated with self-functioning impairments. Mediation analyses indicated that the overall level of personality functioning was consistent with a full mediation model of the ACE–loneliness association, accounting for 64% of the total effect. When examining self-functioning separately, an indirect effect accounted for 78% of the total association. In models testing specific ACE types, self-functioning fully mediated the associations between emotional neglect, sexual violence, and loneliness. Conclusions: The findings highlight self-functioning impairments as a key mechanism linking both cumulative and specific ACEs and loneliness, suggesting a need for a targeted focus on self-related impairments. Longitudinal studies are needed to clarify the developmental trajectories.

## 1. Introduction

Loneliness has emerged as a critical social and public health concern, prompting organizations such as the World Health Organization (WHO) and insurance providers to implement initiatives aimed at understanding its impact to develop effective treatments and interventions [1,2]. Research consistently links loneliness with significant adverse outcomes, including increased risks for mental and physical health disorders, as well as premature mortality [3,4,5,6,7,8]. The relevance of addressing loneliness is further supported by large-scale surveys, which highlight its widespread prevalence. For example, an EU-wide survey [9] reported that 36–40% of respondents regularly experience loneliness, with 12–13% feeling lonely most or all the time [10].

Although demographic and socioeconomic factors are well-established correlates of loneliness, increasing attention has turned toward psychological contributors. Adverse childhood experiences (ACEs), though non-specific, have been linked to a broad range of adult health and psychosocial outcomes, including loneliness [11]. Current research is increasingly seeking to identify the psychological mechanisms by which early adversity may be associated with loneliness in adulthood. This study drew on Criterion A of the Alternative Model for Personality Disorders (AMPD), introduced in Section III of the DSM-5 (Diagnostic and Statistical Manual of Mental Disorders, Fifth Edition) [12], which conceptualizes personality dysfunction dimensionally in terms of impairments in self- and interpersonal functioning. Within this framework, personality functioning provides a theoretical basis for examining its associations with both ACEs and loneliness.

Loneliness is not inherently burdensome. The ability to be alone has been described as a developmental achievement that allows individuals to tolerate and even enjoy solitude [13]. Unlike loneliness, which is a subjective experience resulting from unmet social needs, social isolation is defined by objective indicators such as the number of days spent alone, marital status, or the size of one’s social network [14,15,16]. However, the associations between objective indicators and loneliness are only weak to moderate, meaning that while social isolation contributes to loneliness, it does not fully account for the variance in loneliness [17,18]. When loneliness becomes chronic or severe, individuals often describe it as a deeply distressing and painful experience [3,19].

Recent research on psychological factors and loneliness has examined two key issues [11,20]. The first is the role of adverse childhood experiences (ACEs) as predictors of loneliness. While ACEs are generally associated with an increased risk of several physical and mental health conditions [21,22], meta-analyses and reviews have explored their relationship with loneliness. A recent meta-analysis by de Heer et al. [23] found a moderate effect size, indicating that individuals with a history of maltreatment are more likely to experience loneliness, particularly when the maltreatment involved emotional abuse or neglect [20,24,25]. Similarly, a representative sample of U.S. adults (N = 1839) found that childhood trauma was significantly associated with emotional but not social loneliness [26], with each additional ACE increasing the likelihood of belonging to the loneliness group by 28% [23]. A recent systematic review and meta-analysis by Curtis et al. [27], based on 20 studies and a pooled sample of N = 3255, provided evidence that both cumulative ACE exposure and ACE subtypes are significantly associated with loneliness in adulthood. Multiple adversities frequently co-occur and may exert compounding effects on psychological development. A robust dose–response pattern has been consistently observed, with higher numbers of ACEs associated with greater psychological burdens and loneliness.

The second issue concerns the relationship between loneliness and personality disorders (PDs). In general, individuals with personality disorders report higher levels of loneliness than the general population [24], with social networks that are systematically smaller, less diverse, and less satisfying [26,28]. Reinhard et al. [20] reviewed empirical studies on personality disorders and loneliness, suggesting that specific personality-related factors contribute to this association. They proposed a theoretical model of loneliness in PDs, emphasizing intra- and interpersonal deficits as key mechanisms underlying loneliness. According to this model, intrapersonal factors such as rejection sensitivity, information processing biases, distrust, shame, and self-blame, along with interpersonal factors such as social withdrawal, dysfunctional behaviour, and limited social skills, create a self-reinforcing cycle of loneliness that contributes to the severity and persistence of PDs. Besides the relevance of intra- and interpersonal factors, Reinhard et al. [20] also highlighted the role of adverse childhood experiences (ACEs) as a predisposing factor for both loneliness and personality disorders. In their theoretical model of loneliness, Reinhard et al. [20] underscored the relevance of these dimensions and called for further research to clarify their role in loneliness. Despite these theoretical considerations, no empirical studies have yet directly examined the relationship between loneliness and personality functioning according to Criterion A of the AMPD [12]. Criterion A conceptualizes personality pathology in terms of impairments in self-functioning (identity and self-direction) and interpersonal functioning (empathy and intimacy).

Personality functioning may be a key candidate variable for understanding loneliness for several reasons. First, in current diagnostic manuals, the understanding of personality disorders has been conceptualized dimensionally, spanning a spectrum from no dysfunction to severe dysfunction. The shift from a categorical to a dimensional approach in personality diagnostics has allowed for a more nuanced assessment of self- and interpersonal functioning as transdiagnostic dimensions, meaning they are relevant across multiple mental health conditions rather than being specific to a single disorder [29]. In the Alternative Model of Personality Disorders (AMPD, Criterion A) [12], self-functioning includes identity, which refers to maintaining a stable and coherent sense of self or identity, including self-esteem and a self-concept, and self-direction, which involves setting and pursuing meaningful goals, engaging in self-reflection, and regulating emotions and behaviours. Interpersonal functioning is determined by empathy, the ability to understand and appreciate others’ experiences and perspectives, and intimacy, the capacity to develop and sustain close, mutually satisfying relationships [30]. Beyond personality psychopathology, this dimensional severity perspective also provides valuable insights in the subclinical domain [31], where impairments in self- and interpersonal functioning may contribute to loneliness. Interventions targeting aspects of self- and interpersonal functioning, such as identity stability, self-regulation, empathy, and intimacy, could help prevent difficulties in social relationships and reduce the risk of chronic loneliness.

Second, the shift toward dimensionality in assessing the psychopathology of personality disorders is particularly notable as it contrasts with traditional personality research, which emerged from lexical and factor-analytic models [32,33]. While these approaches have been valuable, they have been criticized for their interpretability issues, as the identified factors may lack clear theoretical underpinnings and clear clinical relevance [34,35,36]. In contrast, the dimensional models of personality disorders in the current DSM-5 [12] and ICD-11 (International Classification of Diseases, 11th Revision) [37] are grounded in longstanding clinical knowledge, integrating insights from object relations theory, mentalization-based approaches, schema therapy, and attachment theory [30]. These models emphasize self- and interpersonal functioning as core components of personality pathology, bridging the gap between empirical research and clinically meaningful conceptualizations [38,39,40].

Third, personality functioning is a relatively recent construct that has gained traction as a key explanatory variable in psychopathology [41,42]. Within the Alternative Model for Personality Disorders (AMPD), self- and interpersonal functioning are conceptualized as internalized representations of the self and others, cognitive–affective structures shaped by early relational experiences [30,43,44,45]. This conceptualization draws on psychodynamic and attachment-based theories, which emphasize the developmental impact of early caregiving on personality organization. Childhood maltreatment has been associated with impairments in the development of personality functioning. Empirical research has indicated that higher ACE exposure is linked to greater impairments in personality functioning [46]. Furthermore, studies examining self- and interpersonal functioning as potential mediators between ACEs and psychological outcomes have found stronger associations for self-functioning, suggesting it may play a more prominent role in this pathway [47].

### Current Study

This study examined whether impairments in personality functioning account for the variance in the association between adverse childhood experiences (ACEs) and loneliness, using a mediation framework within a cross-sectional design. While prior studies have reported separate associations between childhood maltreatment, personality dysfunction, and loneliness, no research has explicitly tested a mediation model or examined whether personality functioning explains a significant portion of the observed association between ACEs and loneliness.

Based on dimensional models of personality pathology and prior findings (e.g., [47,48]), we proposed two hypotheses:

**Hypothesis** **1:** *Greater exposure to ACEs is associated with higher levels of overall personality dysfunction (PF_total_), which in turn is associated with increased loneliness. Thus, we expected that personality functioning statistically mediates the association between ACEs and loneliness*.

**Hypothesis** **2:** *Both self-functioning and interpersonal functioning are hypothesized to mediate the ACE–loneliness association, such that greater ACE exposure is associated with greater impairments in each domain, which in turn are associated with higher loneliness levels. Based on prior findings showing stronger associations for self-functioning than interpersonal functioning in relation to mental health outcomes [47], we expected self-functioning to exhibit a more prominent mediating role. Additionally, we explored whether specific types of ACEs show differential associations with loneliness mediated by these personality functioning domains*.

By testing these hypotheses, this study aimed to offer a more integrated understanding of the patterns consistent with developmental risk models linking early adversity to loneliness, thereby informing future longitudinal research.

## 2. Materials and Methods

### 2.1. Sampling and Study Design

This study was designed as a quantitative, cross-sectional online survey. Data collection was conducted in the German language by using LimeSurvey (LimeSurvey GmbH, Hamburg, Germany) and took place between February and April 2021. The final sample comprised 334 participants, including students at the University of Innsbruck as well as their families and friends, who gave their consent to participate. The students were invited by email using the internal mailing list of the University with a request to forward the survey. Psychology students had the opportunity to receive 1.25 subject hours in accordance with standard departmental procedures for fulfilling research participation requirements. No other incentives were given to other participants. Of the original 518 participants, participants whose data sets had missing or incorrect entries were excluded. The data were collected in a pseudo-anonymized manner, and ethical approval was granted by the Review Board for Ethical Questions of the Institute of Psychology at the University of Innsbruck (N° 34/2020).

### 2.2. Measures

#### 2.2.1. Sociodemographic Data

Participants’ age, gender (female, male, and other), and relationship status were assessed as sociodemographic variables. Their relationship status was assessed using the yes/no question “Are you currently in a partnership?”.

#### 2.2.2. Adverse Childhood Experiences (ACEs)

We adapted the KERF-40-I Stressful Childhood Experiences scale [49] for online administration to reduce participant distress. Seven ACE categories were included: “physical violence”, “verbal violence”, “emotional violence”, “non-verbal emotional violence”, “sexual violence”, “emotional neglect”, and “physical neglect”. Each category was assessed with six binary yes/no items corresponding to distinct age ranges (1–3, 4–6, 7–9, 10–12, 13–15, and 16–18 years). Participants were able to select multiple age ranges for each adversity category, allowing for the assessment of exposure across multiple developmental periods, thereby providing a proxy for the cumulative exposure to adversity. To minimize the risk of emotional overload and potential re-traumatization, we avoided giving explicit examples of violent acts, instead allowing participants to interpret their experiences subjectively.

For the statistical analysis, a total ACE sum score was calculated across all the categories for each participant. Each “yes” answer was awarded 1 point, resulting in a total score of between 0 and 42 per participant. For example, if a participant reported “physical violence” between the ages of 7 and 9, as well as “emotional violence” between the ages of 13 and 15 and again between 16 and 18, this participant would score 3 points. Consistent with standard practice in ACE research [50,51], both cumulative ACE scores and individual ACE categories were analyzed. Different ACE types were treated as conceptually distinct indicators of adversity, rather than assuming a single latent construct, allowing for the examination of both additive and specific effects across trauma subtypes [22,52]. To determine the overall response consistency, we computed Cronbach’s alpha across the 42 binary ACE items (7 categories × 6 age ranges). The resulting internal consistency coefficient was α = 0.93, indicating the high statistical interrelatedness of the reported adversities. However, this value should not be interpreted as evidence of unidimensionality.

#### 2.2.3. Personality Functioning (PF)

Personality functioning was assessed using the German version of the Level of Personality Functioning Scale (LPFS-BF) [53,54]. The 12-item self-report questionnaire measures Criterion A of the AMPD system and showed good internal consistency (α = 0.84). The items (e.g., “I often don’t know who I really am”) are rated on a 4-point Likert scale from 1 (“strongly disagree”) to 4 (“strongly agree”). The LPFS-BF measures two core dimensions of personality functioning: self-functioning (reflecting impairments in identity and self-direction) and interpersonal functioning (reflecting impairments in empathy and intimacy). Each dimension yields a separate subscale score. A total personality functioning score was derived by summing the self- and interpersonal functioning scores, with higher scores indicating greater impairments in personality functioning.

#### 2.2.4. Loneliness

To measure loneliness, we used the UCLA Loneliness Scale in its revised [55] German version [56]. The instrument measures loneliness using 20 items rated on a 4-point Likert scale from 1 (‘never’) to 4 (‘often’). A total of 10 items are positively polarized (e.g., ‘I feel in tune with the people around me’); the other 10 are negatively polarized (e.g., ‘I lack companionship’). The internal consistency of the scale could be described as excellent in our sample (α = 0.93). For the analysis, a mean value was calculated using the scale, with higher values reflecting more loneliness.

### 2.3. Statistical Analysis

Data pre-processing included checking the accuracy of entries, the standardization of variables, and the exclusion of participants with missing or incorrect data, resulting in a final sample of 334 participants. Descriptive statistics and correlations were obtained and multiple linear regressions were conducted using the software IBM SPSS Statistics (version 29.0.1.0, IBM, Armonk, NY, USA). Mediation analysis was performed using IBM SPSS AMOS (version 26.0, IBM, Armonk, NY, USA). RStudio (version R 4.4.2, Posit PBS, Boston, MA, USA) and the “MBESS” package (version 4.9.3) were used to calculate different measures of the effect size of the indirect mediation effect.

As a first step, we calculated the Pearson correlations to assess the presence of significant associations between participants’ age, gender, relationship status, ACEs, personality functioning (total score and self-functioning and interpersonal functioning subscales), and loneliness. The effect sizes were defined according to Cohen [57] as small (r < 0.3), medium (0.3 ≤ r ≤ 0.5), or large (r > 0.5).

To test the associations between ACEs, personality functioning, and loneliness, we conducted several separate multiple linear regressions. Participants’ age, gender, and relationship status were included as covariates. We report standardized beta values (ß) for the regression analyses and the subsequent mediation models. Linear relationships between the predictors and residuals, as well as homoscedasticity, were analyzed using scatter plots. Cook’s distance was used to identify potentially influential data points. The multicollinearity was checked using a tolerance of x < 0.01 and a variance inflation factor (VIF) of x < 10.

For the mediation model, we used AMOS’s maximum likelihood estimators with 5000 bootstrapped samples to define 95% confidence intervals for non-normally distributed data. It is important to note that while mediation models are used in cross-sectional designs to examine how variables may account for the observed associations, this approach does not allow for causal inferences regarding their temporal precedence. ACEs were selected as the independent variable, loneliness as the dependent variable, and personality functioning as the mediator. The covariates included were the same as in the regression analysis. In accordance with the literature [58], a good model fit was defined as an RMSEA (Root Mean Square Error of Approximation) < 0.05 and an SRMR (Standardized Root Mean Residual) < 0.06, with the TLI (Tucker–Lewis Index) and CFI (Comparative Fit Index) preferably being above 0.95. Following Preacher and Kelley [59], we report, in addition to the completely standardized indirect effect, the mediation ratio (PM) as a measure of the relative magnitude of the indirect effect and Κ^2^ as the proportion of the maximum possible indirect effect. The value of Κ^2^ can be interpreted according to Cohen’s [57] guidelines as small (0.01), medium (0.09), or large (0.25). Only mediation models in which the proposed mediator showed an initial association with the independent variable and for which the model fit indices met commonly accepted thresholds were retained and reported.

## 3. Results

### 3.1. Descriptive Statistics (See Table 1)

The sample consisted of N = 334 participants, of whom 217 were female (65.0%), 116 were male (34.7%), and 1 described themselves as other (0.3%). The mean age was 25.96 years (SD = 10.19), and most participants were in a partnership (52.4%), while 47.6% were single. The descriptive statistics and the correlations between all the variables used in this study are shown in Table 1. ACEs showed moderate but highly significant correlations with loneliness and personality functioning (PF_total_) and its self-functioning subscale (PF_self_), but not with the interpersonal subscale (PF_interpersonal_). Additionally, loneliness showed a moderate to large and highly significant association with personality functioning and all of its subscales.

**Table 1:** Descriptive statistics and correlations between age, sex, relationship status, adverse childhood experiences (ACEs), loneliness, and personality functioning (PF_total_, PF_self_, and PF_interpersonal_).

		M	SD	1		2		3		4		5		6		7	
1	Age	25.96	10.19														
2	Gender	0.30	0.95	−0.02													
3	Relationship Status	1.48	0.50	−0.26	***	−0.20	***										
4	ACEs	6.78	7.20	0.03		0.00		0.11	*								
5	Loneliness	1.64	0.50	−0.10		0.04		0.20	***	0.22	***						
6	PF_total_	23.13	5.98	−0.24	***	0.11	*	0.22	***	0.24	***	0.60	***				
7	PF_self_	12.81	4.04	−0.28	***	0.13	*	0.21	***	0.29	***	0.59	***	0.93	***		
8	PF_interpersonal_	10.32	2.69	−0.10		0.05		0.17	***	0.09		0.45	***	0.83	***	0.56	***

Note. M = mean; SD = standard deviation. * *p* < 0.05, *** *p* < 0.001, n = 334. Gender: −1 = male; 1 = female; 0 = other. Relationship status: 1 = in relationship; 2 = not in relationship.

To assess the comparability of our sample, we carried out one-sample t-tests with standardized samples for the results for personality functioning (LPFS) and loneliness (UCLA). We compared the distribution of the number of ACEs in our sample with that in larger cohort studies. In our sample, we found a distribution of 26.9% with zero experienced ACEs, 52,7% with one to three ACEs, and 20,4% with four or more ACEs. Our results are comparable to those from the CDC-Kaiser Permanente ACE study (*n* = 17,337) [60], which reported that 36.1% of participants had 0 ACEs, 51.4% had 1–3 ACEs, and 12.5% had 4 or more ACEs. To formally test the comparability, a chi-square test comparing the ACE category distributions between our sample and the CDC-Kaiser sample was conducted. Although the result was statistically significant (χ^2^(2) = 23.53, *p* < 0.001), the effect size was very small (Cramér’s V = 0.036) The statistical significance likely reflects small but systematic differences, such as a slightly higher proportion of individuals with multiple (four or more) ACEs in our sample, which may be explained by variations in the assessment methods (as we added an extra category for verbal abuse, split the emotional abuse category into two categories, and did not assess household challenges) and the higher proportion of women in our sample.

Comparing the results we obtained for personality functioning (M = 23.13, SD = 5.98) with the norm values reported by Spitzer et al. [54], we found significantly higher personality impairment in our sample when compared to sample 1 [t(333) = 16.73, *p* ≤ 0.001] but no significant difference compared to sample 2 [t(333) = 1.24, *p* = 0.216]. The slightly higher personality impairment in our sample can be explained by the effect of age on the rank correlation demonstrated by Spitzer et al. [54], as our sample was significantly younger. Regarding loneliness (M = 1.64, SD = 0.50), we found no significant difference when comparing our sample to a German norm sample [t(333) = 1.85, *p* = 0.070] [56].

### 3.2. Analysis Strategy

Our analysis was structured as follows: First, we conducted regression analyses to examine the associations between adverse childhood experiences (ACEs) and all the proposed potential mediators (personality functioning: PF_total_, PF_self_, and PF_interpersonal_), as well as the outcome variable (loneliness). In addition, we performed multiple regression analyses in which all seven specific ACE types (e.g., emotional abuse, emotional neglect, physical violence) were included simultaneously as predictors for each outcome variable (PF_total_, PF_self_, PF_interpersonal_, and loneliness). This approach allowed us to assess the unique association of each ACE type with the outcomes while statistically adjusting for the co-occurrence of other adversities. Given the multiple comparisons across seven predictors, we applied a Bonferroni correction, considering associations significant at a threshold of *p* < 0.0071. A correlation table and a description of the results for the individual ACE variables are provided in the Supplementary Materials. In the second step, we conducted additional regression analyses to assess the direct association between personality functioning and loneliness, based on the expectation that higher personality impairments would be positively associated with loneliness. Finally, using a statistical approach within a mediation analysis framework, we explored whether the observed cross-sectional association between ACEs (cumulative ACEs and specific ACE types) and loneliness could be statistically accounted for by an indirect path mediated by personality functioning (total level and subscales) as a potential mechanism linking early adversity to loneliness. Given the cross-sectional design of the study, we interpreted these results as associations consistent with mediation rather than as evidence of causal mechanisms. Throughout our analysis, we report standardized beta coefficients (β) to compare the relative contributions of each predictor to the dependent variable.

#### 3.2.1. Association Between Adverse Childhood Experiences (ACEs), Personality Functioning, and Loneliness (See Table 2, Models A1–A4)

In Model A1, ACEs significantly predicted the overall level of personality functioning (β = 0.23, *p* < 0.001), indicating that a higher number of adverse childhood experiences were associated with greater impairments in personality functioning. This predictor had a small to moderate effect size (f^2^ = 0.06), with the model explaining 14% of the variance in the overall level of personality functioning (R^2^_adj_ = 0.14, *p* < 0.001). Among the covariates, participants’ relationship status (β = 0.17, *p* = 0.002) and gender (β = 0.14, *p* = 0.007) were also significant predictors, with individuals not in a relationship and female participants reporting higher levels of personality impairment. Age was negatively associated with personality impairment (β = −0.20, *p* < 0.001), suggesting that younger individuals exhibited greater personality dysfunction.

In Model A2, ACEs remained a significant predictor of self-functioning impairments (β = 0.29, *p* < 0.001), accounting for 19% of the variance (R^2^_adj_ = 0.19, *p* < 0.001). This suggests that childhood adversity has a more pronounced impact on self-functioning impairments compared to the overall level of personality functioning. Participants’ relationship status (β = 0.14, *p* = 0.007) and gender (β = 0.15, *p* = 0.003) were also significant predictors, while age showed a stronger negative association (β = −0.25, *p* < 0.001), suggesting younger participants experience greater self-functioning impairments.

**Table 2:** Summary of multiple linear regression analysis assessing whether adverse childhood experiences (ACEs) predicted personality functioning (PF_total_, PF_self_, and PF_interpersonal_) and loneliness.

	Model A1 PF_total_				Model A2 PF_self_				Model A3 PF_interpersonal_				Model A4 Loneliness			
	B	SE (B)	β		B	SE (B)	β		B	SE (B)	β		B	SE (B)	β	
Age	−0.11	0.03	−0.20	***	−0.10	0.02	−0.25	***	−0.02	0.02	−0.06		−0.01	0.01	−0.06	
Gender	1.80	0.64	0.14	**	1.27	0.42	0.15	**	0.51	0.31	0.09		0.08	0.06	0.08	
Relationship Status	2.00	0.65	0.17	**	1.13	0.42	0.14	**	0.87	0.31	0.16	**	0.18	0.06	0.18	**
ACEs	0.19	0.04	0.23	***	0.16	0.03	0.29	***	0.03	0.02	0.08		0.01	0.01	0.20	***
R^2^_adj_.	0.14	***			0.19	***			0.03	**			0.08	***		

Note. B = regression coefficient; SE (B) = standard error; β = standardized coefficient; adj. = adjusted. ** *p* < 0.01, *** *p* < 0.001, n = 334.

In Model A3, ACEs were not significantly associated with impairments in interpersonal functioning (β = 0.08, *p* = 0.164), and the effect size was small, explaining only 3% of the variance (R^2^_adj_ = 0.03, *p* = 0.005). This indicates that childhood adversity contributes less to interpersonal functioning impairments compared to self-functioning impairments.

In Model A4, ACEs were significantly associated with loneliness (β = 0.20, *p* < 0.001), suggesting that individuals with greater exposure to childhood adversity experienced higher levels of loneliness. Participants’ relationship status also emerged as a significant predictor (β = 0.18, *p* = 0.002), with those without a romantic partner reporting greater loneliness. However, age (β = −0.06, *p* = 0.267) and gender (β = 0.08, *p* = 0.203) were not significantly associated with loneliness. Overall, the model accounted for 8% of the variance in loneliness (R^2^_adj_ = 0.08, *p* < 0.001).

#### 3.2.2. Association Between Types of Adverse Childhood Experiences (ACEs), Personality Functioning, and Loneliness (See Table 3)

We report here only those associations that remained significant after applying a Bonferroni correction, using a threshold of *p* < 0.0071. Four hierarchical multiple regression models were created to examine the predictors of personality functioning and loneliness. In the model predicting the overall level of personality functioning (PF_total_), age was negatively associated with impairment (*p* < 0.001), indicating that a younger age was related to greater difficulties. Participants’ relationship status was positively associated (*p* = 0.002), with being single linked to higher impairment. Emotional neglect also showed a significant positive association (*p* = 0.002), suggesting that greater exposure was related to more impaired personality functioning overall.

In the model predicting self-functioning (PF_self_), similar patterns emerged: a younger age (*p* < 0.001) and being single (*p* = 0.007) were associated with greater self-functioning impairments. In addition, both sexual violence (*p* = 0.002) and emotional neglect (*p* < 0.001) were significantly linked to higher levels of self-dysfunction. The model predicting interpersonal functioning (PF_interpersonal_) yielded no predictors that met the Bonferroni-corrected significance threshold.

**Table 3:** Associations between types of adverse childhood experiences (ACEs), personality functioning (PF_total_, PF_self_, and PF_interpersonal_), and loneliness.

	PF_total_					PF_self_				
	B	SE (B)	β	*p*		B	SE (B)	β	*p*	
Age	−0.11	0.03	−0.19	0.000	*	−0.10	0.02	−0.24	0.000	*
Gender	0.79	0.34	0.13	0.020		0.54	0.22	0.13	0.014	
Relationship Status	1.99	0.65	0.17	0.002	*	1.15	0.42	0.14	0.007	*
Physical Violence	−0.59	0.29	−0.13	0.042		−0.28	0.19	−0.09	0.131	
Verbal Violence	0.62	0.28	0.18	0.025		0.32	0.18	0.13	0.079	
Emotional Violence	−0.08	0.34	−0.02	0.819		−0.02	0.22	−0.01	0.944	
Non-Verbal Emotional Violence	0.09	0.32	0.03	0.779		0.02	0.21	0.01	0.917	
Sexual Violence	0.94	0.58	0.09	0.107		1.18	0.38	0.16	0.002	*
Emotional Neglect	0.75	0.23	0.21	0.002	*	0.61	0.15	0.25	0.000	*
Physical Neglect	−0.41	0.47	−0.05	0.383		−0.23	0.30	−0.04	0.450	
R^2^_adj_.	0.17					0.23				
	**PF_interpersonal_**					**Loneliness**				
	**B**	**SE (B)**	**β**	***p***		**B**	**SE (B)**	**β**	***p***	
Age	−0.02	0.01	−0.06	0.290		−0.01	0.01	−0.08	0.152	
Gender	0.24	0.16	0.09	0.132		0.03	0.03	0.05	0.349	
Relationship Status	0.84	0.31	0.16	0.007		0.15	0.06	0.15	0.007	
Physical Violence	−0.30	0.14	−0.15	0.028		0.01	0.03	0.01	0.847	
Verbal Violence	0.30	0.13	0.19	0.022		0.02	0.02	0.06	0.442	
Emotional Violence	−0.06	0.16	−0.04	0.703		−0.01	0.03	−0.03	0.781	
Non-Verbal Emotional Violence	0.07	0.15	0.04	0.658		0.02	0.03	0.07	0.477	
Sexual Violence	−0.24	0.28	−0.05	0.389		0.06	0.05	0.06	0.260	
Emotional Neglect	0.14	0.11	0.09	0.206		0.08	0.02	0.26	0.000	*
Physical Neglect	−0.18	0.22	−0.05	0.425		−0.16	0.04	−0.23	0.000	*
R^2^_adj_.	0.05					0.13				

Note. B = regression coefficient; SE (B) = standard error; β = standardized coefficient; adj. = adjusted; * Bonferroni correction threshold of p < 0.0071, n = 334.

In the loneliness model, emotional neglect (*p* < 0.001) was positively associated with loneliness, whereas physical neglect (*p* < 0.001) was negatively associated. This indicates that individuals reporting higher emotional neglect also reported higher loneliness levels, while those reporting higher physical neglect surprisingly reported lower levels of loneliness. Adjusted R^2^ values indicated that the models explained 17% (PF_total_), 23% (PF_self_), 5% (PF_interpersonal_), and 13% (loneliness) of the variance, respectively.

Based on these findings, we conducted additional mediation analyses using only the ACE predictors that remained significant after Bonferroni correction (*p* < 0.0071) to control for multiple testing. Additional mediation models were tested for the following pathways:

(1) Emotional neglect → total level of personality functioning → loneliness.
(2) Emotional neglect and sexual violence → self-functioning → loneliness.

#### 3.2.3. Association Between Personality Functioning and Loneliness (Table 4, Models B1–B2)

To examine the relationship between personality functioning and loneliness, we created two multiple linear regression models (see Table 4). In Model B1, PF_total_ was included as the predictor, along with participants’ age, gender, and relationship status as covariates. The model explained 36% of the variance in loneliness (R^2^_adj_ = 0.36, *p* < 0.001). PF_total_ was significantly associated with loneliness (β = 0.60, *p* < 0.001), indicating that higher personality impairment was related to greater loneliness. However, none of the covariates (age, gender, and relationship status) showed a significant effect (all had a β < 0.10 and *p* > 0.05).

**Table 4:** Summary of multiple linear regression analysis for the prediction of loneliness based on personality functioning (PF_total_, PF_self_, and PF_interpersonal_).

	Model B1 Loneliness				Model B2 Loneliness			
	B	SE (B)	β		B	SE (B)	β	
Age	0.01	0.01	0.06		0.01	0.01	0.08	
Gender	−0.01	0.02	−0.01		−0.01	0.02	−0.02	
Relationship Status	0.09	0.05	0.09		0.09	0.05	0.09	
PF_total_	0.05	0.01	0.60	***				
PF_self_					0.06	0.01	0.51	***
PF_interpersonal_					0.03	0.01	0.15	**
R^2^_adj_.	0.36	***			0.37	***		

Note. B = regression coefficient; SE (B) = standard error; β = standardized coefficient; adj. = adjusted. ** *p* < 0.01, *** *p* < 0.001, n = 334.

In Model B2, PF_self_ and PF_interpersonal_ were included separately, along with the same covariates. This model explained 37% of the variance in loneliness (R^2^_adj_ = 0.37, *p* < 0.001). PF_self_ showed a strong positive association with loneliness (β = 0.51, *p* < 0.001), while PF_interpersonal_ was also significantly associated with loneliness, though with a smaller effect (β = 0.15, *p* = 0.004). Again, none of the covariates (age, gender, relationship status) were significant predictors of loneliness.

These findings suggest that self-functioning impairments play a larger role in loneliness compared to interpersonal functioning impairments. While interpersonal difficulties also contribute to loneliness, their effect appears to be less pronounced.

The full model explained 32% more of the variance in loneliness compared to the null model, indicating that personality functioning contributes substantially to loneliness beyond the effects of demographic factors alone. An ANOVA comparison confirmed that this improvement in the model fit was statistically significant [ΔR^2^ = 0.321, F(1, 329) = 167.80, *p* < 0.001]. The lack of significant effects for participants’ age, gender, or relationship status suggests that personality functioning may be a more relevant predictor of loneliness than these demographic factors.

#### 3.2.4. Mediation Modelling (See Table 5)

Mediation modelling was conducted to explore potential pathways linking adverse childhood experiences (cumulative ACEs and specific types of ACEs) to loneliness (see Table 5). Specifically, we tested whether impairments in the overall level of personality functioning (PF_total_; model series MED1) and self-functioning (PF_self_; model series MED2) mediated the association between cumulative ACEs and specific ACE types and loneliness. As interpersonal functioning was not significantly associated with ACEs and a mediation model including interpersonal functioning did not meet commonly accepted thresholds for an adequate model fit, it was not included as a mediator and is therefore not reported.

**Table 5:** Total, direct, and indirect effects of ACEs on loneliness. Mediator = personality functioning (PF_total_ and PF_self_).

		Std. Total Effect		Std. Direct Effect		Std. Indirect Effect		Κ^2^	P_M_	Result
		Point Est.	95% CI	Point Est.	95% CI	Point Est.	95% CI			
MED1/Mediator: PF_total_										
1.1	ACEs → Loneliness	0.20	[0.09, 0.31]	0.07	[−0.04, 0.17]	0.13	[0.07, 0.20]	0.15	0.64	Full mediation
1.2	Emotional Neglect → Loneliness	0.24	[0.12, 0.36]	0.10	[−0.01, 0.20]	0.14	[0.08, 0.20]	0.16	0.57	Full mediation
MED2/Mediator: PF_self_										
2.1	ACEs → Loneliness	0.20	[0.09, 0.31]	0.03	[−0.08, 0.14]	0.17	[0.10, 0.23]	0.18	0.78	Full mediation
2.2	Emotional Neglect → Loneliness	0.22	[0.10, 0.34]	0.07	[−0.03, 0.17]	0.15	[0.09, 0.22]	/	/	Full mediation
	Sexual Violence → Loneliness	0.07	[−0.10, 0.24]	−0.03	[−0.20, 0.14]	0.10	[0.05, 0.15]	/	/	Full mediation

Note. CI = confidence interval; Std. = standardized; Est. = estimate; n = 348; bootstrapping sample size = 5000. Std. total, direct, and indirect effects were controlled for age, gender, and relationship status. Κ^2^ = proportion of maximum possible indirect effect; PM = mediation ratio. Κ^2^ and P_M_ were calculated using “MBESS” package, excluding covariates.

#### 3.2.5. Model Series MED1: PF_total_ as Mediator (See Figure 1A,B)

**Figure 1:**
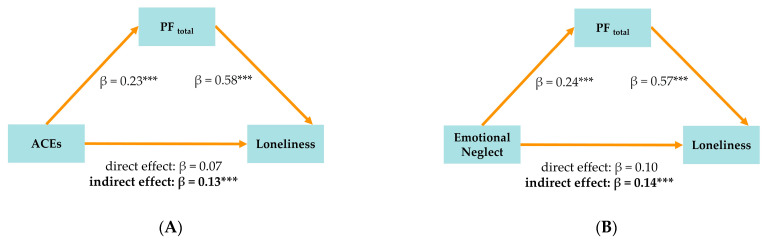
(**A**) MED1.1: Mediation model of PF_total_ showing relationship between adverse childhood experiences (ACEs) and loneliness. *** *p* < 0.001. (**B**) MED1.2: Mediation model of PF_total_ showing relationship between emotional neglect and loneliness. *** *p* < 0.001.

In Model MED1.1 (see Figure 1A), we examined whether impairments in the overall level of personality functioning (PF_total_) mediated the association between ACEs and loneliness. A higher total ACE score was significantly associated with a greater personality functioning impairment (β = 0.23, 95% CI [0.13, 0.32]), which, in turn, was significantly related to increased loneliness (β = 0.58, 95% CI [0.49, 0.66]). The standardized total effect was small but significant (β = 0.20, 95% CI [0.09, 0.31]), with an indirect effect accounting for 64% of the total effect (β = 0.13, 95% CI [0.07, 0.20]). After including PF_total_ in the model, the direct effect of ACEs on loneliness was no longer significant (β = 0.07, 95% CI [−0.04, 0.17]), a finding consistent with full statistical mediation in this model (Κ^2^ = 0.15). Adding PF_total_ to the model substantially increased the explained variance in loneliness from 8% (see Table 2, Model A4, R^2^_adj_.) to 37%.

We then extended this analysis by testing emotional neglect as a specific predictor (Model MED1.2). A higher level of emotional neglect was significantly associated with a greater impairment in personality functioning (β = 0.24, 95% CI [0.12, 0.36]), which, in turn, was significantly associated with increased loneliness (β = 0.57, 95% CI [0.49, 0.66]) (see Figure 1B). The total effect of emotional neglect on loneliness was small but significant (β = 0.24, 95% CI [0.12, 0.36]), and the indirect effect mediated by PF_total_ was statistically significant (β = 0.14, 95% CI [0.08, 0.20]), accounting for the majority of the total effect. After including PF_total_ in the model, the direct effect of emotional neglect on loneliness was no longer significant (β = 0.10, 95% CI [−0.01, 0.20]), supporting full statistical mediation (Κ^2^ = 0.16). Including PF_total_ in this model explained 38% of the variance in loneliness.

#### 3.2.6. Model Series MED2: PF_self_ as Mediator (See Figure 2A,B)

**Figure 2:**
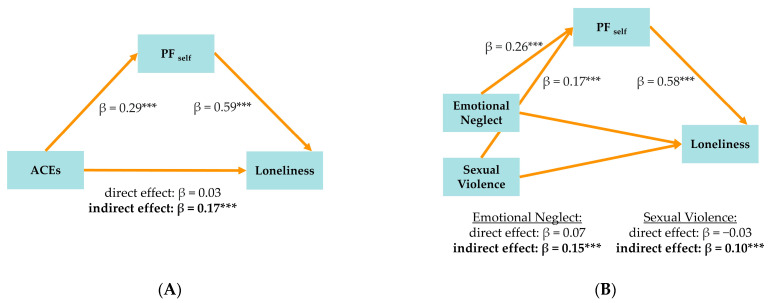
(**A**) MED2.1: Mediation model of PF_self_ showing relationship between adverse childhood experiences (ACEs) and loneliness. *** *p* < 0.001. (**B**) MED2.2: Mediation model of PF_self_ showing relationship between emotional neglect and sexual violence and loneliness. *** *p* < 0.001.

Similarly, in MED2 (see Figure 2A), we examined whether the observed association between ACEs and loneliness supported the hypothesis of a potential pathway involving self-functioning by including PF_self_ in the model. ACEs were significantly related to PF_self_ (β = 0.29, 95% CI [0.18, 0.38]), which, in turn, was associated with increased loneliness (β = 0.59, 95% CI [0.50, 0.66]). The indirect effect was significant and of a medium strength (Κ^2^ = 0.18, β = 0.17, 95% CI [0.10, 0.23]), accounting for 78% of the total effect. Again, the direct effect of ACEs on loneliness was no longer significant (β = 0.03, 95% CI [−0.08, 0.14]) in this model, a finding consistent with full statistical mediation. Including PF_self_ in the model increased the explained variance in loneliness to 36%.

We then extended the analysis by examining emotional neglect and sexual violence as specific predictors in a combined model with PF_self_ as the mediator. Emotional neglect was significantly associated with a greater impairment in self-functioning (β = 0.26, 95% CI [0.15, 0.38]), which, in turn, was associated with increased loneliness (β = 0.58, 95% CI [0.50, 0.66]) (see Figure 2B). The total effect of emotional neglect on loneliness was significant (β = 0.22, 95% CI [0.10, 0.34]), and the indirect effect mediated by PF_self_ was also significant (β = 0.15, 95% CI [0.09, 0.22]), while the direct effect was not significant (β = 0.07, 95% CI [−0.03, 0.17]), indicating full mediation. For sexual violence, the total effect on loneliness was smaller (β = 0.07, 95% CI [−0.10, 0.24]), but the indirect effect via PF_self_ was significant (β = 0.10, 95% CI [0.05, 0.15]), while the direct effect was not significant (β = −0.03, 95% CI [−0.20, 0.14]), again supporting full statistical mediation. Including PF_self_ in the model explained 36% of the variance in loneliness.

#### 3.2.7. Model Fit (See Table 6)

The fit indices for both mediation models indicate a good model fit (see Table 4). The chi-square values suggest no significant model misfits. The RMSEA (0.033–0.038) and SRMR (0.025–0.032) values fell within acceptable thresholds, indicating a low residual error. Additionally, the TLI (0.969–0.978) and CFI (0.990–0.994) values exceeded the 0.95 cutoff, further supporting the adequacy of the models in fitting the observed cross-sectional data [58].

**Table 6:** Fit indices of mediation model series MED1 and MED2.

	X^2^	df	RMSEA	SRMR	TLI	CFI
MED1.1	5.82	4	0.037	0.027	0.972	0.992
MED1.2	5.43	4	0.033	0.025	0.978	0.994
MED2.1	5.88	4	0.038	0.027	0.973	0.993
MED2.2	10.16	7	0.037	0.032	0.969	0.990

Note. n = 334. X^2^ = chi-square; df = Degrees of Freedom; RMSEA = Root Mean Square Error of Approximation; SRMR = Standardized Root Mean Square Residual; TLI = Tucker–Lewis Index; CFI = Comparative Fit Index.

## 4. Discussion

In the present study, using a cross-sectional design, we examined (a) the association between personality functioning measured using total, self-, and interpersonal scores according to Criterion A of the DSM-5 AMPD and loneliness and (b) the mediating role of these personality dimensions in the relationship between cumulative and specific adverse childhood experiences (ACEs) and loneliness, tested in separate models. Although the results must be interpreted with caution due some limitations, our findings align with previous research [47]. The following discussion elaborates on these findings and draws on psychodynamic perspectives to explore potential developmental processes linking early adversity, personality functioning, and loneliness.

### 4.1. Personality Functioning as a Predictor of Loneliness

Our findings replicate and extend previous work by Ernst et al. [48], who also found a significant association between personality functioning and loneliness, though treated loneliness as a mediating rather than primary outcome. However, their study did not differentiate between self- and interpersonal functioning, leaving open the question of whether self- and interpersonal functioning contribute differentially to loneliness. Loneliness is typically described as a subjective experience that arises from a perceived discrepancy between desired and actual social connection. In our study, the overall level of personality functioning was significantly associated with loneliness, with impairments in self-functioning emerging as a more prominent predictor of loneliness compared to the weaker contributions of interpersonal impairments.

According to the AMPD framework, low self-functioning is characterized by an unstable or incoherent identity, chronic feelings of emptiness, fragile self-esteem, and difficulties with emotions and self-regulation. Individuals with low levels of self-functioning may also lack coherent life goals and the capacity for meaningful self-reflection [12,30]. The association between self-functioning and loneliness observed in our data may thus reflect more profound disturbances in self-related functioning, aligning with previous research that has found associations, e.g., between low self-esteem, a diminished sense of mattering, and loneliness [61,62,63,64].

While interpersonal functioning includes capacities such as perspective-taking and the ability to form and maintain reciprocal relationships, self-functioning reflects an individual’s ability to access and express internal states, regulate their affect, and maintain a sense of agency and continuity over time—that is, how individuals experience and understand themselves [12,30]. Impairments in this domain may manifest as an incoherent sense of self, including difficulties forming a stable internal identity or accessing meaningful inner experiences [30], which may in turn contribute to loneliness. Even when interpersonal skills are relatively intact, impaired self-functioning, often reflected in a fragmented or incoherent sense of self, may limit the capacity to feel emotionally connected to others, regardless of the actual social contact or interpersonal success. This may help explain, at least in part, the stronger association we observed between self-functioning and loneliness relative to the weaker role of interpersonal impairments. Nevertheless, this interpretation warrants further empirical investigation.

### 4.2. Personality Functioning as a Mediator Between Childhood Trauma and Loneliness

Our mediation analysis revealed that cumulative adverse childhood experiences were associated with increased loneliness, consistent with previous research [11,24,26]. This relationship was statistically mediated by personality functioning, supporting our hypothesis that impairments in personality functioning may represent a potential central pathway linking early adversity to later loneliness. At the subscale level, only self-functioning mediated the relationship between ACEs and loneliness, suggesting that impairments in self-related domains (e.g., identity coherence, self-direction) may be particularly relevant in understanding how childhood adversity relates to feelings of loneliness. Krakau et al. [65], using a representative German sample, also identified impairments in individuals’ identity perception and self-reflective capacity as key mediators linking childhood maltreatment to both mental and physical health outcomes.

When examining specific ACE subtypes, we found that emotional neglect showed a significant indirect effect on loneliness mediated by the total level of personality functioning. Additionally, both emotional neglect and sexual violence were associated with loneliness through significant indirect effects mediated by self-functioning. Emotional neglect also showed a significant indirect effect mediated by the total level of personality functioning in a separate model. In all cases, the indirect effects were significant, and the direct effects of the ACEs on loneliness became non-significant, indicating indirect-only mediation. These findings underscore the distinct psychological impact of specific ACE types, which appear to undermine the development of core aspects of personality functioning, with emotional neglect affecting both the total level of personality functioning and self-functioning and sexual violence specifically affecting self-functioning. Emotional neglect, characterized by a consistent lack of emotional attunement and responsiveness, may deprive children of the necessary relational context to develop identity coherence, emotional self-awareness, and a sense of agency. Sexual violence, by contrast, constitutes a direct and often traumatic violation of bodily and psychological boundaries and is frequently associated with overwhelming shame, dissociation, and mistrust.

D’Huart et al. [47] similarly found that the overall level of childhood maltreatment, as well as emotional neglect and sexual abuse, predicted mental health outcomes mediated by impaired personality functioning in separate models. However, when both self- and interpersonal functioning were included as potential mediators, only self-functioning significantly mediated the effects of emotional neglect and the overall level of childhood maltreatment. Dagnino et al. [66] similarly found that personality functioning mediated the association between physical and sexual abuse and depressive symptoms, supporting the broader relevance of personality functioning as a potential developmental pathway through which sexual abuse may influence psychological outcomes.

Although the mediation model involving interpersonal functioning did not meet the statistical criteria and this type of functioning only showed a weaker contribution in the regression model, its relevance is supported by theoretical and empirical frameworks. As outlined in the theoretical model of loneliness by Reinhard et al. [20], both self- and interpersonal personality dysfunction contribute to the development and maintenance of loneliness. Interpersonal impairments—such as social withdrawal, reduced perspective-taking, and difficulties with empathy, intimacy, or mutuality—may limit the ability to sustain reciprocal relationships, respond flexibly to relational challenges, or accurately interpret others’ intentions. Prior research has linked loneliness to poor social skills [28], including specific interpersonal deficits such as social withdrawal [67] and reduced empathy or mutuality [68]. These difficulties may contribute to social exclusion and chronic loneliness. However, future research using longitudinal designs and more representative samples is needed to better understand the role of interpersonal personality functioning in the development and persistence of loneliness.

Taken together, our findings suggest that self-functioning may represent a potential psychological pathway through which early relational adversity—particularly emotional neglect and sexual violence—is associated with later experiences of loneliness. The consistent pattern of indirect-only effects in the mediation models, along with the stronger predictive role of self-functioning in the regression analyses, underscores the relevance of self-related capacities such as identity integration, emotional self-awareness, and emotion regulation (AMPD, DSM-5). These capacities may be important for interpreting one’s emotional and social experiences, and their impairment could contribute to enduring feelings of disconnection. This pattern is consistent with prior research identifying personality functioning as a core transdiagnostic factor associated with a range of psychological and somatic outcomes [46,69,70].

The inclusion of the total level of personality functioning in our models may support its potential clinical utility as a dimensional severity indicator. In current diagnostic frameworks such as the AMPD, personality pathology is understood as existing along a continuum from no dysfunction to severe dysfunction. Total PF scores may thus offer a useful global index of personality impairment that complements the more specific insights provided by scores in the self and interpersonal subdomains. However, future research should investigate potential pathways more systematically, ideally using longitudinal and multi-method designs to clarify temporal and causal processes. In particular, the role of interpersonal functioning as a potential mediator should be further examined.

### 4.3. A Dimensional Personality Perspective to Understanding Loneliness

Our findings contribute to the growing evidence that personality functioning, particularly self-functioning based on the results of our study, plays a central role in understanding loneliness [20]. The Alternative Model of Personality Disorders (AMPD) conceptualizes self- and interpersonal functioning as two core dimensions of personality pathology, existing along a spectrum from subclinical to severe dysfunction [20]. This dimensional approach allows for a more nuanced understanding of loneliness, even in individuals without diagnosed personality disorders. Rather than focusing solely on symptom-based diagnoses, the AMPD has introduced a mental representational level drawing on psychodynamic theory, conceptualizing self- and interpersonal functioning as cognitive–affective structures (self–other representations) that organize the inner psychic world and guide how individuals interpret social interactions, regulate emotions, and form expectations based on their early relationships [71].

Personality functioning largely develops in early childhood and may serve as a key pathway linking adverse childhood experiences (ACEs) to later psychopathology [47,65,66]. Our finding that emotional neglect and sexual violence were associated with loneliness, mediated by self-functioning, supports this view, suggesting that early relational trauma can disrupt core self-capacities. This interpretation aligns with previous research showing that impairments in personality functioning, particularly in the self domain, mediate the relationship between childhood adversity and later psychological outcomes [47,65]. When this development is impaired, individuals may form maladaptive schemas—such as “I am lonely” or “I am unlovable”—that may embed loneliness into the personality structure itself. Fonagy et al. [72] emphasize that trauma is especially harmful when experienced in a state of “subjective aloneness,” in which one’s internal experience is not mentalized or contained by another mind. This form of relational trauma may lie at the core of persistent loneliness.

Kernberg’s model [73,74] of early object-relational failure, consistent with principles of mentalization theory, illustrates how early relational disruptions can lead to structural impairments in personality functioning. Emotionally unavailable or inconsistent caregiving may disrupt the integration of self–other representations and the development of mentalizing capacities, leading to identity diffusion and the use of primitive defences such as splitting. These vulnerabilities have been described as manifesting in chronic feelings of emptiness, disconnection, and interpersonal dysfunction, which may in fact reflect deeper disturbances in the self [72]. This form of “structural loneliness” is not defined by the absence of others, but by the absence of a coherent internal self that can be experienced as stable and meaningful, both to oneself and within close relationships.

From a clinical perspective, these insights underscore the importance of therapeutic relationships that support the development of a more coherent and integrated sense of self. Psychodynamic and mentalization-based therapies, including approaches such as Transference-Focused Psychotherapy [75] and Mentalization-Based Treatment [76], in particular, aim to repair deficits in self-functioning by offering a relational context in which internal experiences are mirrored, validated, and gradually integrated. Such interventions may be especially important for addressing the persistent loneliness that arises not from external disconnection but from internal fragmentation. Facilitating a deeper understanding of how personality functioning develops over time in response to early relational experiences—thereby enabling the construction of a coherent narrative of one’s personal development and of how one has become who one is—may be central in restoring self-continuity and alleviating the profound, persistent disconnection that characterizes loneliness.

## 5. Limitations

This study has several limitations that should be acknowledged. First, due to the cross-sectional design, causal inferences could not be made. Longitudinal studies are needed to further clarify the temporal sequence and potential causal pathways linking ACEs, personality functioning, and loneliness.

Second, our sample was not representative, consisting primarily of young adults (mean age of ≈ 26 years), with a majority of female participants (65%) and a substantial proportion of psychology students, whose elevated psychological mindedness and self-awareness may have influenced their self-report responses. This limits the generalizability of the findings to broader populations, including older adults, clinical groups, and individuals outside academic settings. Future studies should aim to replicate these findings using larger and more demographically diverse or representative samples.

Third, ACEs were assessed using an adapted version of the KERF-40-I modified for online use to minimize the participant burden and emotional distress. While this approach allowed for assessment across multiple developmental periods—providing a proxy for cumulative exposure—it also introduced potential limitations, including recall bias, underreporting, and variability in interpretation. These concerns are inherent in the retrospective self-report methodologies widely used in ACE research. Furthermore, the adapted measure has not yet been validated against standardized instruments (e.g., the ACE Questionnaire), which limits the comparability with existing studies and should be addressed in future research.

Fourth, personality functioning was assessed using the 12-item LPFS-BF. Although this instrument demonstrates good internal consistency and is widely used in both clinical and non-clinical populations—as well as in online settings such as in the present study—it may not fully capture the complexity of constructs like identity coherence or intimacy. In addition, our reliance solely on self-report measures raised the possibility of shared method variance, which may have inflated the observed associations. As noted, future research should incorporate multi-method approaches (e.g., clinician ratings, peer or informant reports) and replicate these findings using interview-based assessments and more diverse samples to enhance the measurement validity and generalizability.

Finally, future research should examine additional mediators and covariates—such as emotion regulation, depression, perceived and objective social support, or genetic predispositions—to better elucidate the relationship between ACEs, personality functioning, and loneliness. Importantly, such analyses should be conducted within longitudinal frameworks to mitigate the risk of introducing bias through collider adjustment. As this was the first study to examine these associations within the AMPD Criterion A framework, future work would benefit from using longitudinal designs, representative samples, and multi-method assessments (e.g., structured interviews, clinician ratings) to enhance our theoretical understanding and the generalizability of the findings.

## Data Availability

The raw data supporting the conclusions of this article will be made available by the authors without undue reservation.

## Supplementary Materials

The following supporting information can be downloaded at https://www.mdpi.com/article/10.3390/brainsci15060551/s1: Table S1: Descriptive statistics and correlations between cumulative adverse childhood experiences (ACEs) and specific ACE types, loneliness and personality functioning (PF_total_, PF_self_ and PF_interpersonal_).
