# Supplementary material for: The Key Role of Personality Functioning in Understanding the Link Between Adverse Childhood Experiences and Loneliness: A Cross-Sectional Mediation Analysis
Source: Brain Sci. 2025 May 23;15(6):551. doi: 10.3390/brainsci15060551 (PMC12191166; doi:10.3390/brainsci15060551)
Supplement: Supplementary file 1 [file brainsci-15-00551-s001.zip › brainsci-3595088-supplementary.pdf]

Supplementary Material

Bivariate correlations among adverse childhood experiences (ACEs), loneliness, and personality functioning variables are presented in *Table S1*. Total ACE exposure was positively associated with loneliness ($r = .50$, $p < .001$), overall personality functioning impairment ($r = .24$, $p < .001$), self-functioning impairments ($r = .29$, $p < .001$), and interpersonal functioning impairments ($r = .09$, $p = .08$). While the correlation between ACEs and interpersonal functioning was positive, it did not reach statistical significance. Loneliness showed significant positive associations with impairments in overall personality functioning ($r = .60$, $p < .001$), self-functioning ($r = .59$, $p < .001$), and interpersonal functioning ($r = .45$, $p < .001$).

Among specific ACE types, physical and verbal violence, emotional violence, and non-verbal emotional violence also showed significant positive correlations with both self- and overall personality functioning impairments (r ranging from .20 to .34, all $p < .001$). Emotional neglect ($r = .30$, $p < .001$) and sexual violence ($r = .27$, $p < .001$) were notably associated with impairments in self-functioning. Only verbal violence revealed a significant correlation with interpersonal functioning ($r = .13$, $p < .05$).

Regarding loneliness and specific ACE types, emotional neglect ($r = .26$, $p < .001$), sexual violence ($r = .15$, $p < .01$), emotional violence ($r = .19$, $p < .001$), and non-verbal emotional violence ($r = .17$, $p < .01$) were significantly associated with increased loneliness. In contrast, physical violence, verbal violence, and physical neglect showed no significant correlations with loneliness.

Table S1. Descriptive statistics and correlations between cumulative adverse childhood experiences (ACEs) and specific ACE types, loneliness and personality functioning (PF_{total}, PF_{self} and $PF_{interpersonal}$).

		M	*SD*	1	2	3	4	5	6	7	8	9	10	11
1	Physical Violence	.86	1.35											
2	Verbal Violence	1.89	1.72	.56 ***										
3	Emotional Violence	1.56	1.72	.51 ***	.72 ***									
4	Non-Verbal Emotional Violence	1.20	1.67	.48 ***	.66 ***	.80 ***								
5	Sexual Violence	.19	.56	.30 ***	.28 ***	.26 ***	.29 ***							
6	Emotional Neglect	.95	1.67	.44 ***	.48 ***	.56 ***	.56 ***	.30 ***						
7	Physical Neglect	.15	.73	.32 ***	.26 ***	.34 ***	.39 ***	.18 ***	.41 ***					
8	ACEs	6.78	7.20	.71 ***	.83 ***	.88 ***	.86 ***	.42 ***	.76 ***	.50 ***				
9	Loneliness	1.64	.50	.14 *	.19 **	.17 **	.18 **	.15 **	.26 ***	-.08	.22 ***			
10	PF_{total}	23.13	5.98	.07	.22 ***	.20 ***	.20 ***	.18 **	.26 ***	.06	.24 ***	.60 ***		
11	PF_{self}	12.81	4.04	.13 *	.24 ***	.23 ***	.23 ***	.27 ***	.32 ***	.10	.29 ***	.59 ***	.93 ***	
12	$PF_{interpersonal}$	10.32	2.69	-.03	.13 *	.09	.10	-.01	.10	-.02	.09	.45 ***	.83 ***	.56 ***

Note. M = mean, SD = standard deviation. * $p < .05$, ** $p < .01$, *** $p < .001$, n = 334. Gender: -1 = male, 1 = female, 0 = divers. Relationship Status: 1 = with relationship, 2 = without relationship.
